# Lack of LTβR Increases Susceptibility of IPEC-J2 Cells to Porcine Epidemic Diarrhea Virus

**DOI:** 10.3390/cells7110222

**Published:** 2018-11-21

**Authors:** Tawfeek Altawaty, Lulu Liu, Hongyong Zhang, Cong Tao, Shaohua Hou, Kui Li, Yanfang Wang

**Affiliations:** 1 Institute of Animal Science, Chinese Academy of Agricultural Sciences, Beijing 100193, China; tawfeek@foxmail.com (T.A.); liululu2090@126.com (L.L.); taocong@caas.cn (C.T.); houshaohua@caas.cn (S.H.); likui@caas.cn (K.L.); 2 Department of Animal Science, Chinese Agricultural University, Beijing 100193, China; 3 State Key Laboratory of Reproductive Biology, Institute of Zoology, Chinese Academy of Sciences, Beijing 100101, China; zhanghongyong846@163.com

**Keywords:** lymphotoxin beta receptor (LTβR), intestinal porcine enterocyte cells, CRISPR/Cas9, porcine epidemic diarrhea virus (PEDV) infection

## Abstract

The essential requirement of the lymphotoxin beta receptor (LTβR) in the development and maintenance of peripheral lymphoid organs is well recognized. Evidence shows that LTβR is involved in various cellular processes; however, whether it plays a role in maintaining the cellular function of intestinal porcine enterocytes (IPEC-J2), specifically during porcine epidemic diarrhea virus (PEDV) infection, remains unknown. In this study, we generated LTβR null IPEC-J2 cells using CRISPR/Cas9 to examine the importance of LTβR in cell proliferation, apoptosis, and the response to PEDV infection. Our results showed that the lack of LTβR leads to significantly decreased cell proliferation, potentially due to S phase arrest in LTβR^−/−^ IPEC-J2 cells. Label-free digital holographic microscopy was used to record the three-dimensional morphology of both cell types for up to 72 hours and revealed significantly increased numbers of LTβR^−/−^ cells undergoing apoptosis. Furthermore, we found that PEDV-infected LTβR^−/−^ null IPEC-J2 cells exhibited significant suppression of nuclear factor kappa-light-chain-enhancer of activated B cells (NFκB) target genes (interleukin (IL)-6 and IL-8) and mucosal barrier integrity-related genes (vascular cell adhesion molecule 1 (VCAM1) and IL-22), which may explain why LTβR^−/−^ cells are more susceptible to PEDV infection. Collectively, our data not only demonstrate the key role of LTβR in intestinal porcine enterocytes, but also provide data for the improved understanding of the cellular response to PEDV infection.

## 1. Introduction

Lymphotoxin beta receptor (LTβR) belongs to the tumor necrosis factor (TNF) receptor superfamily (TNFRSF), which includes more than 25 receptors that interact with nearly 20 ligands to regulate the immune response, and is activated by the proinflammatory cytokines lymphotoxin α_1_β_2_ or TNF superfamily member 14 (TNFSF14, also named as LIGHT) [1]. LTβR is expressed on the surface of most cell types, with the highest expression on cells of epithelial and myeloid lineages [2].

The critical roles of LTβR in the development and maintenance of peripheral lymphoid organs were illustrated in LTβR-deficient mice two decades ago [3]. Over the past twenty years, LTβR signaling has been reported to be crucially involved in many cellular processes and molecular events. The activation of LTβR by functional antibodies induces nuclear factor kappa-light-chain-enhancer of activated B cells (NFκB)-dependent interleukin-6 (IL-6) and macrophage inflammatory protein-2 (MIP-2) secretion in mouse fibrosarcoma cells [4]. Signaling by lymphotoxin α_1_β_2_ via LTβR is an essential prerequisite for the containment of intracellular pathogens, as LTβR knockout mice exhibit significantly higher numbers of *Mycobacterium tuberculosis* (*M. tuberculosis*) in infected lungs, and these genetically modified mice are substantially more susceptible than wild-type mice to intraperitoneal (i.p.) infection with *Listeria monocytogenes* [5]. In addition to the critical role of LTβR in protection against infection, the involvement of LTβR in the regulation of the microbial community composition has been reported [6]. Specifically, LTβR knockout mice are resistant to high-fat-diet induced obesity and exhibit an overgrowth of segmented filamentous bacteria (SFB) due to lacking IL-22 and IL-23 [6]. Recent studies have found the link between LTβR signaling and oncogenic protein kinase B, also named as AKT, in hepatitis and liver tumorigenesis, demonstrating that the activation of LTβR rapidly accelerates the intrahepatic cholangiocarcinoma progression initiated by the AKT/Notch signaling pathway [7]. Furthermore, single nucleotide polymorphisms (SNPs) in LTβR have been reported to be associated with the spontaneous resolution of hepatitis B virus (HBV) infection in a Chinese population [8].

Recently, conditional knockout mouse models were employed to reveal novel cellular functions of LTβR. The impacts of LTβR on lymph node (LN) development and the vascular LN microenvironment were revealed by endothelial cell-specific LTβR knockout mice, and this study identified endothelial cells as an important LTβR-dependent lymphoid tissue organizer [9]. In addition, it has been demonstrated that LTβR signaling in intestinal epithelial cells is essential for epithelial IL-23 production and protection against epithelial injury [10]. The study of macrophage/neutrophil LTβR-specific knockout mice, which were generated by the flox/LysM-cre system, suggested that LTβR activation on macrophages by the T-cell derived lymphotoxin α_1_β_2_ controls proinflammatory responses via the tripartite-motif protein 30α (TRIM30α) pathway to protect against exacerbating inflammatory reactions [11].

Porcine epidemic diarrhea virus (PEDV) replicates efficiently in the small intestine [12], and PEDV infection causes acute, severe atrophic enteritis, including mild to severe watery diarrhea, dehydration, and vomiting in pigs. Severe outbreaks of PEDV infections were reported in China in 2010 [13] and in North America in 2013 [14], leading to high mortality among infected piglets and huge economic losses. Epithelial cells provide the first line of defense against mucosal pathogens, and IPEC-J2 cells and LTβR signaling in intestinal epithelial cells are required for the recruitment of neutrophils to the site of infection during early infection via the production of the chemokine (C-X-C motif) ligand 1 (CXCL1) and CXCL2 [15]. However, the importance of LTβR in the regulation of PEDV infection in IPEC-J2 cells is currently unknown. In this study, we generated LTβR knockout cells using the CRISPR/Cas9 technique and investigated the effect of LTβR on IPEC-J2 cell proliferation, cell cycle and apoptosis. More specifically, the impact of LTβR on PEDV infection in IPEC-J2 cells was also investigated. 

## 2. Materials and Methods

### 2.1. Porcine Intestine Samples

Porcine gut tissues, including the duodenum, jejunum, ileum, appendix, colon, rectum, and lymph nodes, were collected from four adult male Large White pigs (*n* = 4). All experiments involving animals were performed according to the procedures approved by the Institutional Animal Care and Use Committee of the Institute of Zoology, Chinese Academy of Sciences (CAS) (Ethic approval number: IOZ20160047).

### 2.2. Cell Culture

African green monkey kidney cells (Vero E6) were kept in Shaohua Hou’s laboratory from the Institute of Animal Science (IAS), Chinese Academy of Agricultural Sciences (Beijing, China) and IPEC-J2 cells were purchased from Jennio Biotech Co., Ltd. (Guangzhou, China). Both cells were cultured in Dulbecco’s Modified Eagle’s Medium (DMEM, Gibco BRL, Grand Island, NY, USA) supplemented with 15% fetal bovine serum (FBS, HyClone, Logan, UT, USA) and 1% penicillin–streptomycin. Both cell types were incubated at 37 °C with 5% CO_2_. The Vero cell-adapted PEDV CV777 strain, kept in Hou’s lab from IAS, was propagated as previously described [16].

### 2.3. Gene Targeting by the CRISPR/CAS9 System

The pX330 vector was used to construct the targeting genomic sequences, which were created by the laboratory of Feng Zhang and obtained from Addgene (plasmid 42230). Guide RNAs were designed using an online tool provided by Feng Zhang’s Laboratory at the MIT/BROAD Institute as described previously [17]. The L1 and L3 small guide RNA (sgRNA) genome targeting sequences, (L1: 5′-GGGAATGGCGGGCCTCTTGGTTT-3′; L3: 5′-GAAGGTGCTCCCTTACCGCCCGG-3′), were cloned into the pX330 vector as previously described [16]. IPEC-J2 cells were transfected by nucleofection using an Amaxa^TM^ Nucleofector^TM^ Kit (Lonza, Cologne, Germany). The pCAG-GFP plasmid was cotransfected with the pX330 plasmid as an indicator for fluorescence activated cell sorting (FACS). Twenty-four hours after transfection, cells were subjected to FACS sorting based on the expression of enhanced green fluorescent protein (EGFP) fluorescence. Single cells were plated into each well of 96-well plates and cultured for approximately 10 days in cell culture medium supplemented with 2.5 ng/mL basic fibroblast growth factor (Sigma, St. Louis, MO, USA). The medium was replaced every 3 days. Confluent cell colonies were propagated and subjected to PCR-restriction fragment length polymorphism (RFLP) assays. PCR products (411 bp) were digested with *Aci*I (New England Biolabs, Ipswich, MA, USA) and the restriction fragments were analyzed on a 2% agarose gel. The identified biallelic mutant clones were subjected for sequencing analysis.

### 2.4. Reverse Transcription PCR (RT-PCR)

Two different RT-PCRs were used in this study, real-time PCR and semi-quantitative PCR. Total RNA from tissues and cells was isolated by TRIzol reagent, and RNA concentrations were determined with a NanoDrop apparatus (NanoDrop Technologies, Wilmington, DE, USA). Two milligrams of total RNA was reverse transcribed using a First Strand cDNA Synthesis Kit (Thermo Fisher Scientific, Waltham, MA, USA). Real-time PCR was performed using SYBR Green master mix (Applied Biosystems, Foster City, CA, USA) and a 7500 Fast Real Time PCR system (Applied Biosystems, Foster City, CA, USA). Expression levels were normalized to those of the housekeeping gene, glyceraldehyde-3-phosphate dehydrogenase (GAPDH). Primers used for real-time PCR are shown in Table 1. The relative gene expression was calculated using the comparative cycle threshold (2^−DDCt^) method. The parameter for semi-quantitative PCR was 4 min at 94 °C followed by 26 cycles of 45 s at 94 °C, 30 s at 60 °C, 45 s at 72 °C and a final extension of 5 min at 72 °C. PCR products (10 µL) were used to detect the expression.

### 2.5. Western Blotting

Cells were washed twice with cold phosphate-buffered saline (PBS), and lysate samples were prepared in 350 μL T-PER Tissue Protein Extraction Reagent (Thermo Scientific Pierce, Rockford, IL, USA) in the presence of a protease inhibitor cocktail (Roche, Indianapolis, IN, USA) and centrifuged at 20,000× *g* for 20 min at 4 °C. 

Proteins (20–50 μg) and protein markers were separated by SDS-polyacrylamide electrophoresis in 10% polyacrylamide slab gels and transferred to polyvinylidene difluoride (PVDF) membranes (Millipore, Madison, WI, USA). Blots were blocked in 5% milk in 0.1% Tris-buffered saline-Tween 20 (TBST) for 1 h at room temperature. Then, blots were incubated with antibodies against LTβR (1:1000, Abcam, Cambridge, MA, USA) and β-actin (1:2000, CST, Danvers, MA, USA) overnight at 4 °C. Immunoreactive bands were detected using Pierce enhanced chemiluminescence (ECL) Western Blotting Substrate (Thermo Scientific Pierce, Rockford, IL, USA). 

### 2.6. Cell Proliferation

To examine cell proliferation, LTβR^+/+^ and LTβR^−/−^ cells were plated in 96-well plates at 5 × 10^3^ cells per well in 100 µL cell culture medium and maintained at 37 °C in a humidified incubator containing 5% CO_2_. Proliferation was analyzed every 24 h with the Cell Counting Kit-8 (CCK-8 kit, Beyotime Biotechnology, Shanghai, China) following the manufacturer’s protocol. 

### 2.7. Cell Cycle Analysis 

LTβR^+/+^ and LTβR^−/−^ cells were plated in 6-well plates and serum-starved overnight for synchronization. The next day, serum was added to the cells and after 24 h of stimulation, cells were trypsinized and fixed in cold 70% ethanol. Cells were then incubated at 4 °C for a minimum of 45 min to a maximum of overnight. Subsequently, they were centrifuged at 1500× *g* for 10 min at 4 °C and re-suspended in 0.4% propidium iodide (PI: containing 50 μg/mL propidum iodide with 100 μg/mL RNase A) for staining. Cells were then analyzed with an LSR II cytometer (BD Biosciences, San Diego, CA, USA), and the PI staining intensity was determined by ModFit software (BD Biosciences, San Diego, CA, USA). This analysis gave the percentage of cells in the G1, S, and G2 phases.

### 2.8. Digital HoloMonitor Microscopy

HoloMonitor M4 Microscopy (Phase Holographic Imaging AB, Lund, Sweden) is an imaging time-lapse cytometer based on holographic microscopy, providing imaging and quantification of unstained living cells directly in their culture vessels. LTβR^+/+^ and LTβR^−/−^ cells were seeded in 6-well plates and monitored for 72 h. Apoptosis was analyzed by Hstudio M4 Tracking software (Scheelevägen, Sweden).

### 2.9. Statistical Analysis

Statistical analyses were performed with GraphPad Prism 5.0 (GraphPad software, La Jolla, CA, USA). An unpaired *t*-test was used to compare values (means ± standard error means (SEMs)) between wild-type and mutant cells as described in the figure legends. * *p* < 0.05, ** *p* < 0.01 and *** *p* < 0.001 were considered statistically significant. 

## 3. Results

### 3.1. LTβR Is Highly Expressed in Porcine Gut Tissues

**Figure 1:**
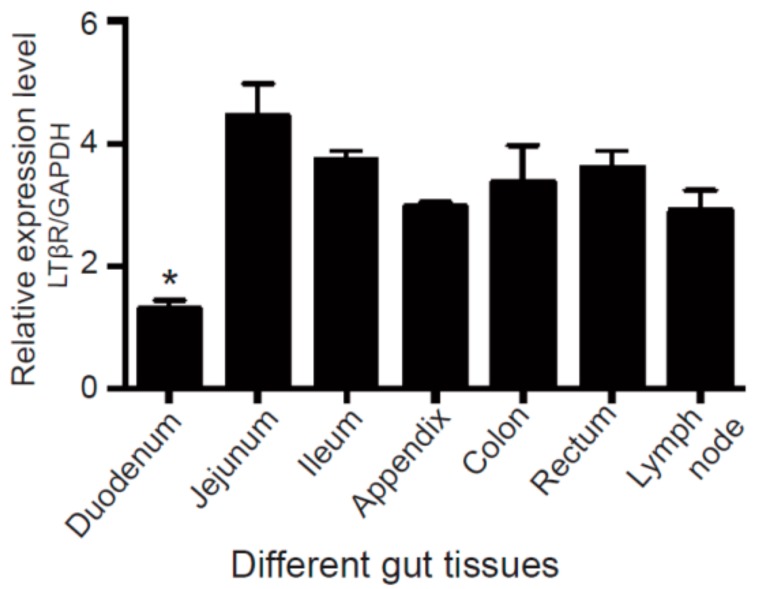
Expression of the lymphotoxin beta receptor (LTβR) in different porcine gut tissues. Tissues, including the duodenum, jejunum, ileum, appendix, colon, rectum and lymph nodes, were collected from adult male Large White pigs (*n* = 4), and real-time PCR was used to measure the expression level of LTβR. * *p* < 0.05 was considered statistically significant. Note that the duodenum showed significantly lower expression of LTβR than other gut tissues.

We first examined expression profiles of *LTβR* in different sections of gut tissues, including the duodenum, jejunum, ileum, appendix, colon, rectum, and lymph nodes, by real-time PCR. Our data showed that LTβR was expressed in all sections of gut tissues and lymph nodes from adult Large White pigs. The highest expression level of LTβR was observed in the jejunum, whereas the duodenum exhibited significantly lower expression compared to other gut sections (Figure 1).

### 3.2. Generation of LTβR^−/−^ Cells Using CRISPR/Cas9

**Figure 2:**
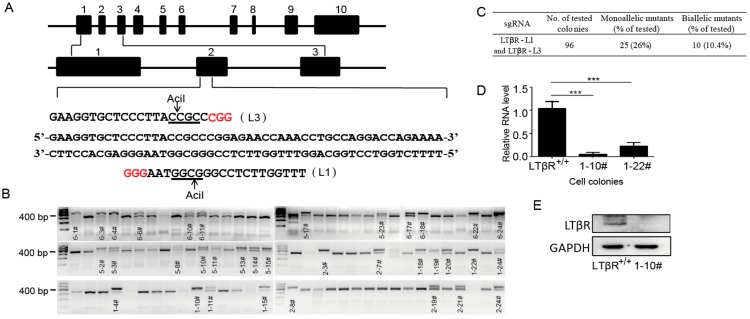
Generation and identification of LTβR knockout intestinal porcine enterocyte (IPEC-J2) cells. (**A**) Targeting strategy: two different small guide RNAs (sgRNAs) (L1 and L3) targeting 32 bp regions of exon 2 in the pig LTβR gene were designed and inserted into the pX330 vector. Then, the pCAG-GFP plasmid was co-transfected with the pX330-L1 and pX330-L3 plasmids, and single cells were sorted into 96-well plates by flow cytometry. (**B**) A total of 96 colonies were picked and subjected to RFLP analysis. Monoallelic and biallelic mutant clones were labeled by their clone number. (**C**) Estimation of targeting efficiency. Note that 10 biallelic mutated clones were identified, and the targeting efficiency was 10.4%. (**D**) Wild-type IPEC-J2 cells and two biallelic mutated clones (namely 1-10# and 1-22#) were picked for real-time PCR analysis. *** *p* < 0.001 was considered statistically significant. Note that the expression levels of LTβR were significantly decreased in 1-10# and 1-22# cells (*p* < 0.001 respectively). (**E**) LTβR protein levels in the wild-type and 1-10# cells were detected by immunoblotting with an anti-LTβR antibody at a 1:1000 dilution (Abcam). GAPDH was used as a loading control.

To generate LTβR knockout IPEC-J2 cells, we designed two different sgRNAs (L1 and L3) that target 32 bp regions in exon 2 of the porcine LTβR gene (Figure 2A). The pCAG-GFP plasmid was co-transfected with the pX330-L1 and pX330-L3 plasmids, and single cells were sorted into 96-well plates by flow cytometry. To determine CRISPR-CAS9-mediated mutations, 96 colonies were selected and subjected to RFLP analysis (Figure 2B). Our data showed that 10 cell clones were biallelically mutated, and the targeting efficiency was 10.4% (Figure 2B,C). To further validate the biallelic mutation, five cell clones, 1-10#, 1-19#, 1-22#, 2-3# and 6-18#, were randomly selected for DNA sequencing (Supplementary Figure S1), and the results confirmed those of RFLP. Further, the amino acid sequences from the wild-type 1-10# cell clone were compared, and our results demonstrated the shifted mutation in both alleles (Supplementary Figure S2).

To examine whether CRISPR/Cas9-mediated gene editing could generate LTβR null alleles in IPEC-J2 cells, we randomly selected two biallelic mutation clones (1-10# and 1-22#) and compared their LTβR expression levels to those of wild-type IPEC-J2 cells (hereafter designated LTβR^+/+^) by real-time PCR. Our data showed that the expression level of LTβR was significantly decreased in both 1-10# and 1-22# (Figure 2D). In addition, the inactivation of LTβR in clone 1-10# was further confirmed by Western blotting (Figure 2E). The non-detectable LTβR expression in 1-10# at both the RNA and protein levels suggested that LTβR was successfully knocked out and these cells are hereafter referred to as LTβR^−/−^ cells and used for the following studies.

### 3.3. LTβR Knockout Inhibits IPEC-J2 Cell Proliferation

**Figure 3:**
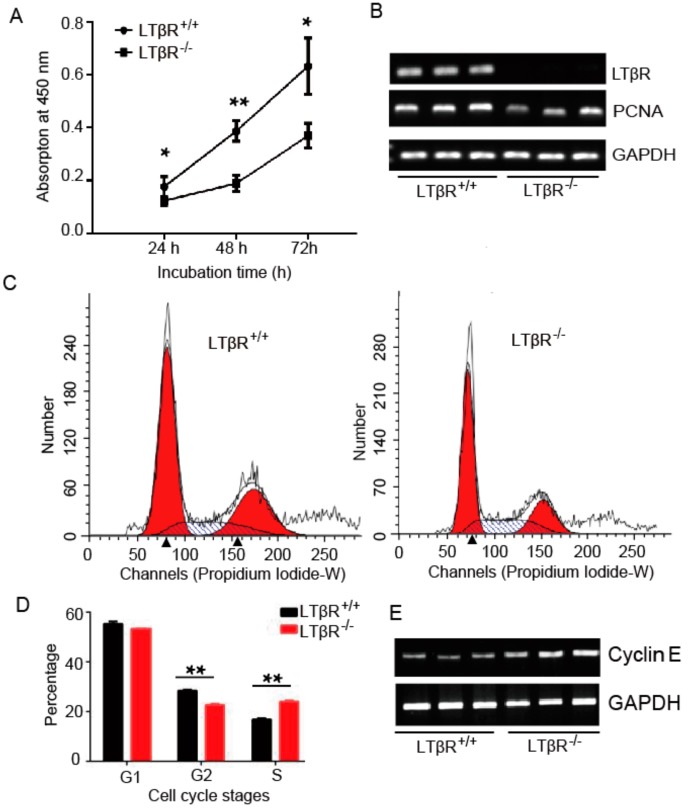
The effect of LTβR on IPEC-J2 cell proliferation and cell cycle progression. (**A**) LTβR^+/+^ and LTβR^−/−^ cells were plated and cultured for various times, and the number of proliferating cells was quantified by the Cell Counting Kit-8 (CCK-8) method. The results shown are representative of three different experiments * *p* < 0.05 and ** *p* < 0.01. (**B**) Semi-quantitative PCR of LTβR and proliferating cell nuclear antigen (PCNA) in both cells. (**C**) LTβR^+/+^ and LTβR^−/−^ cells were serum-starved for 24 h and stimulated with serum. After 24 h, the DNA content of incorporated propidium iodide was scanned on a flow cytometer. Each scan is derived from a representative experiment, where the data from at least 10,000 events were obtained. (**D**) Cell populations in G1, S, and G2 were determined using ModFit software (BD Biosciences, San Diego, CA, USA), ** *p* < 0.01. (**E**) Semi-quantitative PCR of Cyclin E in both cells.

To examine the potential effect of LTβR on cell proliferation, a CCK-8 kit was used to analyze cell proliferation in both LTβR^+/+^ and LTβR^−/−^ cells. As shown in Figure 3A, the in vitro proliferation of LTβR^−/−^ cells was significantly inhibited at 48 h (0.387 ± 0.023 vs. 0.189 ± 0.018 for LTβR^+/+^ and LTβR^−/−^ cells, respectively, *p* < 0.01) and 72 h (0.633 ± 0.062 vs. 0.370 ± 0.027 for LTβR^+/+^ and LTβR^−/−^ cells, respectively, *p* < 0.05), suggesting the ablation of LTβR inhibited IPEC-J2 cell proliferation. In addition, semi-quantitative PCR was used to detect the expression level of proliferating cell nuclear antigen (PCNA), a cell proliferation marker. Consistently, semi-quantitative PCR results showed that PCNA was down-regulated in LTβR^−/−^ cells (Figure 3B). These results suggest that the knockout of LTβR reduces cell growth in vitro.

Since cell growth is tightly regulated by a series of regulators of the cell cycle [18], the effects of LTβR on cell cycle progression were analyzed by flow cytometry using propidium iodide staining. Our data demonstrated S phase arrest in LTβR^−/−^ cells, which resulted in a significant population increase of S phase cells (16.715% ± 0.345 vs. 24.09% ± 0.045 for LTβR^+/+^ and LTβR^−/−^ cells, respectively, *p* < 0.01) and a dramatic decrease of G2 phase cells (28.12% ± 0.33 vs. 22.74% ± 0.36 for LTβR^+/+^ and LTβR^−/−^ cells, respectively, *p* < 0.01) (Figure 3C,D). Cell cycle progression is regulated by a complex network of cell cycle-related genes [19]. Since LTβR^−/−^ cells were arrested at the S phase, the expression of cyclin E1, a key gene in the G1 to S phase transition, was measured by semi-quantitative PCR. The results revealed substantially higher expression levels of cyclin E1 in LTβR^−/−^ cells than in LTβR^+/+^ cells (Figure 3E).

### 3.4. LTβR Knockout Induces IPEC-J2 Cell Apoptosis

**Figure 4:**
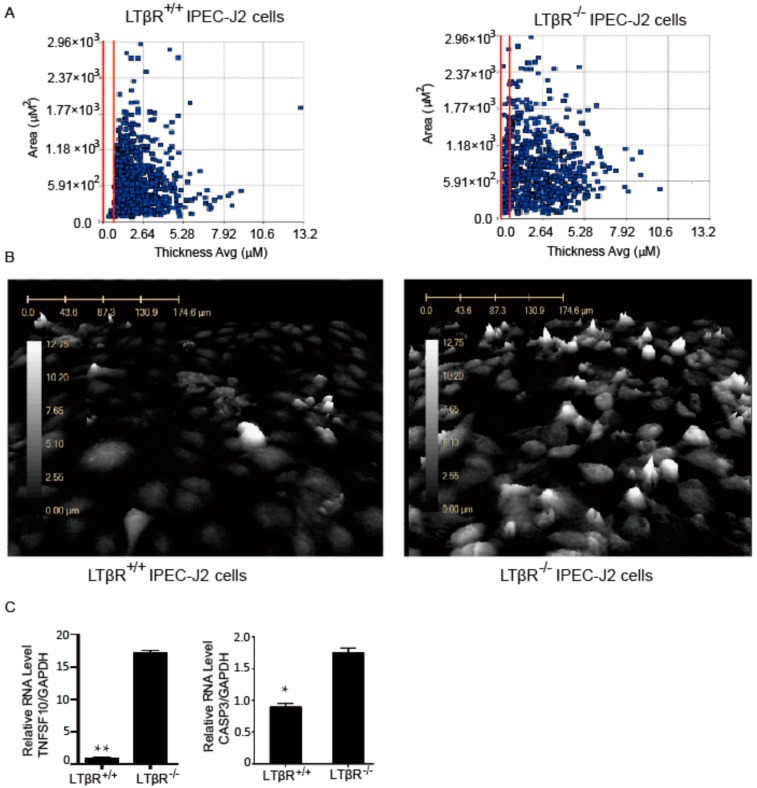
The effect of LTβR on IPEC-J2 apoptosis. LTβR^+/+^ and LTβR^−/−^ cells were seeded into 6-well plates, and apoptosis was monitored for 72 h by HoloMonitor^®M4^ microscopy. (**A**) Dot plot of LTβR^+/+^ and LTβR^−/−^ IPEC-J2 cells after 72 h culture. The x-axis is the average thickness (µM) of the cells, and the y-axis presents the surface area of the cells (µM^2^). Apoptotic cells with lower thickness and higher surface area are between the red lines. Note there are almost no apoptotic cells between the red lines for the LTβR^+/+^ group (left), while significantly more apoptotic cells are found in the LTβR^−/−^ cell population (right). (**B**) Three-dimensional structures of living LTβR^+/+^ (left) and LTβR^−/−^ (right) cells after 72 h. The number of tall cells is noticeably increased in LTβR^−/−^ cells. (**C**) Real-time PCR data of TNF superfamily member 10 (TNFSF10) and Caspase 3 (CASP3) in LTβR^+/+^ and LTβR^−/−^ cells, * *p* < 0.05 and ** *p* < 0.01.

Digital holographic microscopy offers an advantage in studying real-time observations of critical events by exhibiting a continuous two-dimensional (2D) and 3D visual picture of cellular activity in second intervals. A large portfolio of quantitative morphological parameters, including optical cell volume, thickness, area, irregularity, eccentricity, and single-cell tracking, can be recorded and analyzed [20]. Here, digital holographic microscopy was used to monitor dynamic activities and morphological changes of LTβR^+/+^ and LTβR^−/−^ cells in real-time for up to 72 hours. The results showed that these cells displayed distinct growth characteristics. Specifically, many more LTβR null cells than LTβR^+/+^ cells exhibited increased cell volume (vertical axis) and decreased cell membrane thickness (horizontal axis) (Figure 4A, cells between the red lines). Figure 4B shows the 3D structures of observed living LTβR^+/+^ cells (Figure 4B, left) and LTβR^−/−^ cells (Figure 4B, right). Clearly, LTβR^−/−^ cells with a white color are apoptotic because the flow of liquids through apoptotic cell membranes (permeability malfunction) leads to increased cell volume, and the lightened cell membranes therefore reflect different colors.

Next, because we found that the knockout of LTβR induces apoptosis, we investigated apoptosis-related genes in both types of cells. q-PCR was performed to examine the expression levels of apoptosis-related genes, including TNF superfamily member 10 (TNFSF10) and Caspase 3 (CASP3), in both cell lines. Our data revealed that these two genes were significantly increased in LTβR^−/−^ cells (Figure 4C).

### 3.5. LTβR Knockout IPEC-J2 Cells Are Susceptible to PEDV 

**Figure 5:**
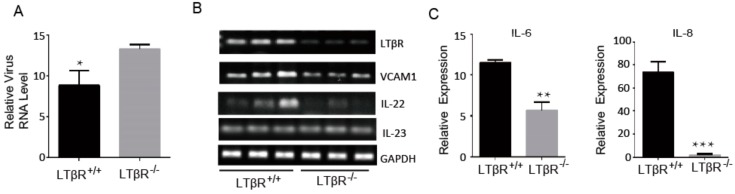
Effect of LTβR on porcine epidemic diarrhea virus (PEDV) infection of IPEC-J2 cells. (**A**) LTβR^+/+^ and LTβR^−/−^ cells were challenged with PEDV CV777 strain (at a multiplicity of infection of 1), and cells were harvested 48 h later to determine relative virus level by real-time PCR using PEDV-specific primers. Note that significantly lower numbers of virus are observed in LTβR^−/−^ cells (* *p* < 0.05). (**B**) Semi-quantitative PCR was performed for LTβR downstream genes in virus infected LTβR^+/+^ and LTβR^−/−^ cells, including vascular cell adhesion molecule 1 (VCAM1), IL-22 and IL-23. Note that LTβR^−/−^ cells had decreased expression of VCAM1 and IL-22. (**C**) Quantitative PCR data of two nuclear factor kappa-light-chain-enhancer of activated B cells (NFκB) targets, IL-6 and IL-8, in virus-infected cells. Note that LTβR^−/−^ cells displayed significantly lower expression of IL-6 and IL-8 (** *p* < 0.01 and *** *p* < 0.001).

LTβR is reportedly highly expressed in the lymph nodes, duodenum and jejunum of eight-day-old newborn piglets, which may be beneficial for developing resistance to *E. coli* F18 in pigs [21], and IPEC-J2 cells are susceptible to PEDV infection [22]. As such, we investigated the effects of LTβR on PEDV infections in IPEC-J2 cells. We challenged LTβR^+/+^ and LTβR^−/−^ cells with the PEDV CV777 strain (at a multiplicity of infection (MOI) of 1), and cells were harvested 48 h later to determine the relative viral expression level by real-time PCR using PEDV-specific primers. Our data showed that RNA levels of PEDV in LTβR^−/−^ IPEC-J2 cells were significantly higher than those in wild-type IPEC-J2 cells (Figure 5A). Since PEDV infection destroys epithelial barrier integrity [22], and LTβR signaling was reported to limit mucosal damage through the IL-22-IL-23 pathway [10], we examined the expression levels of LTβR downstream genes by semi-quantitative PCR, including VCAM1, IL-22 and IL-23, in PEDV-infected cells. Our data revealed that VCAM1 and IL-22 expression was substantially decreased in LTβR^−/−^ IPEC-J2 cells, while IL-23 was intact (Figure 5B). The NFκB-dependent genes IL-6 and IL-8 are reported to be induced by activation of LTβR signaling [4,23]. We detected expression levels of IL-6 and IL-8 in infected cells, and the data showed that both genes were significantly decreased in infected LTβR^−/−^ IPEC-J2 cells (Figure 5C).

## 4. Discussion

LTβR plays a key role in lymphoid organogenesis; however, increasing studies show that LTβR signaling is involved in many cellular processes. Herein, we showed that LTβR is ubiquitously expressed in all sections of gut tissues from adult pigs, indicating its potential function in the intestine. Specifically, LTβR expression was significantly lower in the duodenum than in other intestinal sections. Therefore, we used IPEC-J2 cells, a non-transformed, non-tumorigenic porcine cell line derived from jejunal intestinal regions, to investigate the effects of LTβR on cell proliferation, apoptosis and viral infection.

The blockade of LTβR can be achieved either by using the functional inhibitor LTβR-IgG or by congenital deletion of LTβR or lymphotoxin α1β2. In this study, we successfully generated LTβR null cells using the CRISPR/Cas9 technique, as evidenced by the low expression at both the mRNA and protein levels. The gene targeting efficiency was determined, and the biallelic mutated clone reached 10.4%. Further, our data showed that cell proliferation was significantly inhibited in LTβR null cells, suggesting that LTβR-mediated signaling is required to maintain IPEC-J2 cell proliferation. In agreement with this result, the increased viability of LIGHT-stimulated human bone marrow-derived mesenchymal stem cells (BM-MSCs) was observed [24]. Cell proliferation is closely associated with the cell cycle. We performed cell cycle analysis with both cell lines using propidium iodide staining, and the ablation of LTβR prevents cells from completing the G2/S phase transition, so the cells in the S phase significantly increased. Consistent with these results, expression levels of the S phase marker cyclin E1 were high in LTβR null cells. From these results, we speculate that LTβR knockout prevents cells from exiting the S phase, which leads to growth inhibition in IPEC-J2 cells.

It has been demonstrated that LTβR plays an important role in cell death via caspase-dependent and -independent pathways [25,26]. In this study, we video monitored LTβR^+/+^ and LTβR^−/−^ cells for up to 72 h by digital holographic microscopy. Compared to other strategies that measure cell death and apoptosis, such as the terminal deoxynucleotidyl transferase dUTP nick end labeling (TUNEL) assay, digital holographic microscopy allows the visualization of real-time morphological alterations in cells and has become a powerful tool for the evaluation of cell responses to various stimuli with no labeling required [20]. The 3D images allowed the visualization of apoptotic cells, and the number of tall cells was increased in the LTβR^−/−^ cell population, indicating that more LTβR^−/−^ cells than LTβR^+/+^ cells underwent apoptosis. Furthermore, we observed that the apoptotic-related genes TNFSF10 and CASP3 were significantly increased in LTβR^−/−^ cells, further confirming that the knockout of LTβR induced the apoptosis of IPEC-J2 cells. Interestingly, in contrast from our observations, Wu et al. reported that the overexpression of either LTβR or the cytoplasmic domain of LTβR induces apoptosis in HeLa cells [26]. This opposite observation might be due to the distinct role of LTβR in different cell types.

LTβR signaling has been reported to be crucially involved in many cellular processes and molecular events, and we are particularly interested in its role in bacterial and viral infection. Several reports have demonstrated the critical role of LTβR signaling in bacterial infection of intestinal epithelial cells. For example, LTβR^−/−^ mice are sensitive to bacterial infection due to the absence of lymphoid organs in these mice, and LTβR signaling in intestinal epithelial cells is required for the recruitment of neutrophils to the site of infection during early infection via the production of CXCL1 and CXCL2 [6,27]. LTβR signaling is required for clearance of *Salmonella typhimurium* in infected gut lumen [28]. Additionally, very recent data showed that LTβR is highly expressed in the lymph nodes, duodenum and jejunum of eight-day-old newborn piglets, which may be beneficial for developing resistance to *E. coli* F18 in pigs [21]. In addition, the role of LTβR in viral infection was investigated. Zhu et al. reported that the LTβR rs12345 polymorphism is related to the spontaneous resolution of hepatitis B virus infection [8]. All these observations revealed the function of LTβR in infection. Since IPEC-J2 cells have been reported to be susceptible to PEDV infection [22], we investigated the impact of LTβR on PEDV infection in IPEC-J2 cells. Our data revealed that the levels of PED virus in LTβR^−/−^ cells was significantly higher than in LTβR^+/+^ cells, indicating that LTβR-mediated signaling plays a key role in protecting IPEC-J2 cells from PEDV infection.

Since it has been demonstrated that PEDV infection destroys epithelial barrier integrity [22] and LTβR signaling limits mucosal damage through the IL-22–IL-23 pathway [10], we detected expression levels of VCAM1, IL-22 and IL-23 in both cell types by semi-quantitative PCR. VCAM1 and IL-22 were significantly decreased in RNA expression in LTβR^−/−^ IPEC-J2 cells, in agreement with the previous observation that an abundance of IL-22 is significantly reduced in LTβR^−/−^ mice fed a high-fat-diet [6]. IL-22, a member of the IL-10 superfamily, plays essential roles in fighting against mucosal microbial infection and maintaining mucosal barrier integrity within the intestine. The downregulation of IL-22 and VCAM1 in LTβR^−/−^ IPEC-J2 cells indicates that the epithelial barrier integrity of cells might be impaired, which leads to increased susceptibility to PEDV infection, though further investigation is needed. Surprisingly, IL-23 was found to be intact in LTβR^−/−^ cells, which suggested that IL-22 and IL-23 may play individual roles in IPEC-J2 cells. In addition, the S-phase arrest might also contribute to the higher viral production in LTβR^−/−^ cells. The overexpression of LTβR, or the activation of its mediated signaling by its functional antibody or cellular receptors such as LIGHT, will also be needed to positively determine its role in preventing infection. 

In summary, this study explored the effect of LTβR on IPEC-J2 proliferation and apoptosis, as well as its role in PEDV infection. The absence of LTβR increased susceptibility to PEDV infection in IPEC-J2 cells, which might be caused by significantly suppressed NFκB target genes (IL-6 and IL-8) and mucosal barrier integrity-related genes (VCAM1 and IL-22). Our in vitro cellular model will be helpful for better understanding the biological function of LTβR and the cellular responses to PEDV infection.

## Figures and Tables

**Table 1:** Primers used in this study.

Gene Name	Forward (5′-3′)	Reverse (5′-3′)
Lymphotoxin beta receptor (LTβR)	CACTCATGCTGGGCCTCT	GAGCAGCAGACGTGATGTTT
Vascular cell adhesion molecule 1 (VCAM1)	ATCCAAGCTGCTCCAAAAGA	GGCCCTGTGGATGGTATATG
Interleukin-22 (IL-22)	TTGCTCAAGTTCGTGTCGTC	GGTCAAGCTTGCAGTGATGA
Interleukin-23 (IL-23)	TAGGGGTCGAGTCAGAGGTG	GAGTGCCATCCTTGAGCTGT
Interleukin-6 (IL-6)	CCACCGGTCTTGTGGAGTTT	AGTCGGGTTGTCTAGGCTGA
Interleukin-8 (IL-8)	TGCAAGCTTTGTTATGCAGTG	GCCTGGTGAATTTTTGCTGT
Proliferating cell nuclear antigen (PCNA)	GATTCCACCACCATGTTCGAG	GATTCCACCACCATGTTCGAG
Caspase 3 (CASP3)	GCCATGGTGAAGAAGGAAAA	GTCCGTCTCAATCCCACAGT
Tumor necrosis factor Superfamily member 10 (TNFSF10)	ACCCAAAGGCTCAACAC	CCCACCTGAGATGGATCACT
Glyceraldehyde 3-phosphate dehydrogenase (GAPDH)	GTGAAGGTCGGAGTGAACG	CTCGCTCCTGGAAGATGGTG
Porcine epidemic diarrhea virus (PEDV)	GCACTTATTGGCAGGCTTTGT	CCATTGAGAAAAGAAAGTGTCGTAG

## Supplementary Materials

The following are available online at http://www.mdpi.com/2073-4409/7/11/222/s1, Figure S1: Sequencing analysis of cell clones 1-10#, 1-19#, 1-22#, 2-3# and 6-18#, Figure S2: Amino acid sequences alignment for wild-type, 1-10# and 1-19# cell clones.
