# Supplementary material for: Lack of LTβR Increases Susceptibility of IPEC-J2 Cells to Porcine Epidemic Diarrhea Virus
Source: Cells. 2018 Nov 21;7(11):222. doi: 10.3390/cells7110222 (PMC6262443; doi:10.3390/cells7110222)
Supplement: Supplementary file 1 [file cells-07-00222-s001.pdf]

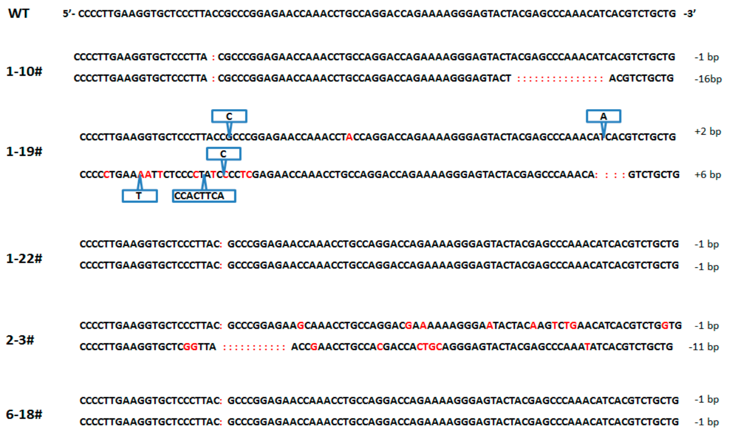

Supplementary Figure 1. To validate the biallelic mutation that we screened using the CRISPR/Cas9 technique, the cell clones 1-10#, 1-19#, 1-22#, 2-3# and 6-18# were randomly selected for DNA sequencing.

WT----MRLPWAASSCGLAWGPLMLGLCSLQAASQSQLVLPYCPENQTCQDQKREYYEPKHHVCCSRCPP

allele1----**M**RLPWAASSCGLAWGPL**M**LGLCSLQAASQSQLVLPYARRTKPARTRKGSTTSPNITSAARAAPQ

allele2----**M**RLPWAASSCGLAWGPL**M**LGLCSLQAASQSQLVLPYARRTKPARTRKGSTTS......... AARAAPQ

Supplementary Figure 2. Amino acid sequences from the wild-type (1-10#) and 1-19# cell clones were compared, and our results demonstrate the shifted mutation in both alleles.

Supplementary Table 1

Primers used in this study

Gene name	Forward (5′-3′)	Reverse (5′-3′)
LTβR	CACTCATGCTGGGCCTCT	GAGCAGCAGACGTGATGTTT
VCAM1	ATCCAAGCTGCTCCAAAAGA	GGCCCTGTGGATGGTATATG
IL-22	TTGCTCAAGTTCGTGTCGTC	GGTCAAGCTTGCAGTGATGA
IL-23	TAGGGGTCGAGTCAGAGGTG	GAGTGCCATCCTTGAGCTGT
IL-6	CCACCGGTCTTGTGGAGTTT	AGTCGGGTTGTCTAGGCTGA
IL-8	TGCAAGCTTTGTTATGCAGTG	GCCTGGTGAATTTTTGCTGT
PCNA	GATTCCACCACCATGTTCGAG	GATTCCACCACCATGTTCGAG
CASP3	GCCATGGTGAAGAAGGAAAA	GTCCGTCTCAATCCCACAGT
TRAIL	ACCCAAAGGCTCAACAC	CCCACCTGAGATGGATCACT
GAPDH	GTGAAGGTCGGAGTGAACG	CTCGCTCCTGGAAGATGGTG
